# Validation of an automated contouring and treatment planning tool for pediatric craniospinal radiation therapy

**DOI:** 10.3389/fonc.2023.1221792

**Published:** 2023-09-22

**Authors:** Soleil Hernandez, Hester Burger, Callistus Nguyen, Arnold C. Paulino, John T. Lucas, Austin M. Faught, Jack Duryea, Tucker Netherton, Dong Joo Rhee, Carlos Cardenas, Rebecca Howell, David Fuentes, Julianne Pollard-Larkin, Laurence Court, Jeannette Parkes

**Affiliations:** ^1^ The University of Texas MD Anderson Cancer Center UTHealth Houston Graduate School of Biomedical Sciences, Houston, TX, United States; ^2^ Department of Radiation Physics, The University of Texas MD Anderson Cancer Center, Houston, TX, United States; ^3^ Department Medical Physics, Groote Schuur Hospital and University of Cape Town, Cape Town, South Africa; ^4^ Department of Radiation Oncology, The University of Texas MD Anderson Cancer Center, Houston, TX, United States; ^5^ Department of Radiation Oncology, St. Jude Children’s Research Hospital, Memphis, TN, United States; ^6^ Department of Radiation Oncology, University of Alabama at Birmingham, Birmingham, AL, United States; ^7^ Department of Imaging Physics, The University of Texas MD Anderson Cancer Center, Houston, TX, United States; ^8^ Department of Radiation Oncology, Groote Schuur Hospital and University of Cape Town, Cape Town, South Africa

**Keywords:** autoplanning, autocontouring, pediatrics, global health, radiation oncology

## Abstract

**Purpose:**

Treatment planning for craniospinal irradiation (CSI) is complex and time-consuming, especially for resource-constrained centers. To alleviate demanding workflows, we successfully automated the pediatric CSI planning pipeline in previous work. In this work, we validated our CSI autosegmentation and autoplanning tool on a large dataset from St. Jude Children’s Research Hospital.

**Methods:**

Sixty-three CSI patient CT scans were involved in the study. Pre-planning scripts were used to automatically verify anatomical compatibility with the autoplanning tool. The autoplanning pipeline generated 15 contours and a composite CSI treatment plan for each of the compatible test patients (n=51). Plan quality was evaluated quantitatively with target coverage and dose to normal tissue metrics and qualitatively with physician review, using a 5-point Likert scale. Three pediatric radiation oncologists from 3 institutions reviewed and scored 15 contours and a corresponding composite CSI plan for the final 51 test patients. One patient was scored by 3 physicians, resulting in 53 plans scored total.

**Results:**

The algorithm automatically detected 12 incompatible patients due to insufficient junction spacing or head tilt and removed them from the study. Of the 795 autosegmented contours reviewed, 97% were scored as clinically acceptable, with 92% requiring no edits. Of the 53 plans scored, all 51 brain dose distributions were scored as clinically acceptable. For the spine dose distributions, 92%, 100%, and 68% of single, extended, and multiple-field cases, respectively, were scored as clinically acceptable. In all cases (major or minor edits), the physicians noted that they would rather edit the autoplan than create a new plan.

**Conclusions:**

We successfully validated an autoplanning pipeline on 51 patients from another institution, indicating that our algorithm is robust in its adjustment to differing patient populations. We automatically generated 15 contours and a comprehensive CSI treatment plan for each patient without physician intervention, indicating the potential for increased treatment planning efficiency and global access to high-quality radiation therapy.

## Introduction

Each year, 300,000 children are diagnosed with cancer worldwide. Of these, 90% live in low- and middle-income countries (LMICs), where access to proper care may be limited by available resources (1). Globally, the 5-year survival rate for patients with pediatric cancer has increased to over 80% in high-income countries (HIC); however, this trend has not been mirrored in LMICs, where average survival rates remain as low as 20% in some countries (2). Recognizing this issue, the World Health Organization launched the Global Initiative for Childhood Cancer (GICC) program in 2018 aiming to increase global survival from pediatric cancer to 60% (3). Radiation therapy is complex and time-consuming to plan and deliver, yet it plays a critical role in managing cancer in more than 50% of pediatric patients in LMICs, and its use is expected to rise to 78% over the next 10 years (4).

Pediatric brain and CNS tumors constitute the leading cause of deaths associated with pediatric cancer world-wide (5), but even more so in LMICs where access to diagnosis and treatment requires availability of technical and human resources (6). Medulloblastoma is the most common malignant brain tumor in children accounting for 20-25% of pediatric malignancies in HICs with large variations in incidence in LMICs. Patients with this diagnosis (as well as some other pediatric brain tumors) require craniospinal radiotherapy, one of the most technically demanding techniques in a radiotherapy center (7, 8).

Limited personnel create demanding workflows. For example, medical physicists dedicate up to 50% of their time to generating radiation therapy treatment plans (9). To alleviate demanding workflows and increase global access to high-quality radiation therapy, artificial intelligence has been introduced to automate various aspects of the radiation therapy treatment planning process. The Radiation Planning Assistant (RPA) planning team has developed algorithms to automate contouring, treatment planning, and quality assurance for adult disease sites, including the cervix, chest wall, spine, head and neck, and whole brain (10–15). Court et al. recently summarized how the RPA was designed alongside leaders in resource-constrained countries to address the global expertise gap in radiation oncology (16). In short, clinicians import a patient CT scan with a planning prescription into the RPA webpage. The web-based servers of the RPA then automatically generate contours and a corresponding treatment plan using internal algorithms. The contour and plan files are then sent back to the user for download. The RPA was developed with clinical acceptability and safety/risk in mind to ensure successful deployment, and increase global access to high-quality radiation therapy.

Recently, as part of the RPA project, Hernandez et al. introduced artificial intelligence into pediatric radiation oncology to facilitate autosegmentation and planning for craniospinal radiation therapy for pediatric patients with medulloblastoma (17). In addition, Hernandez et al. investigated automatically contouring postoperative GTV volumes using a pediatric dataset (18). Both studies were exclusively trained, validated, and tested on an internal pediatric dataset.

The performance of deep learning models has been shown to decrease when tested on patient populations from different hospitals often due to heterogeneity in medical imaging techniques (19). In addition, models trained only on a single dataset may be susceptible to overfitting, which may further limit the generalizability of the model on different patient populations (20). Chen et al. reported that one of the biggest challenges of incorporating artificial intelligence–based tools into radiation oncology is the generalizability of deep learning models (21). In 2021, the FDA recognized that artificial intelligence may be biased towards the dataset it is tested on. In outlining strategies to mitigate bias in algorithm development, it was highlighted that the algorithms should be tested on diverse patient cohorts to test generalizability (22).

To evaluate the generalizability of our algorithms, we tested our CSI autocontouring and autoplanning tool developed at our institution, on a large dataset from another institution. We recruited three pediatric radiation oncologists from three different institutions to comprehensively evaluate the performance of the autocontouring and autoplanning tool. Automating the contouring and planning workflow for pediatric CSI has the potential to increase access to high-quality radiation therapy, as time saved in treatment planning may be allocated to other clinically necessary tasks.

## Methods

We tested the CSI autocontouring tool on a dataset from St. Jude Children’s Research Hospital, comprising of 63 full-body CSI CT scans. This study was approved by our institutional review board. The dataset was curated such that each patient had been previously treated with photons in the head-first-supine position. Of the 63 scans, 30 had been performed on Siemens machines and 33 had been performed on Philips machines. The median (range) number of slices, slice thicknesses, and tube voltage peaks were 495 (225–780), 1.5 (1–3) mm, and 120 (120–120) kVp, respectively. After evaluating the imaging parameters, all CT images were imported into the Raystation treatment planning system version 11B (Raysearch Laboratories, Stockholm, Sweden) (23).

### Autocontouring

Two deep-learning based autosegmentation pipelines were employed to generate the normal tissue contours on the 63 CT scans outside of the treatment planning system. Deep learning uses a series of multi-layer neural networks to learn image features of large training datasets (image and contour pairs) to then automatically segment contours on independent test datasets (images only). To generate the contours in this study, first, a previously validated, adult head and neck autocontouring model was run to generate the brain, brainstem, eye, lens, and cochlea contours (24). Next, a previously validated, pediatric-specific autocontouring model was used to generate the cribriform plate, lacrimal gland, pituitary gland, thyroid, heart, lung, shoulder, mandible, spinal canal, vertebral column, and kidney contours (17). The inputs of both algorithms are a CT scan, and the outputs are a set of autocontours which may then be imported into the treatment planning system for planning.

### Autoplanning

**Figure 1:**
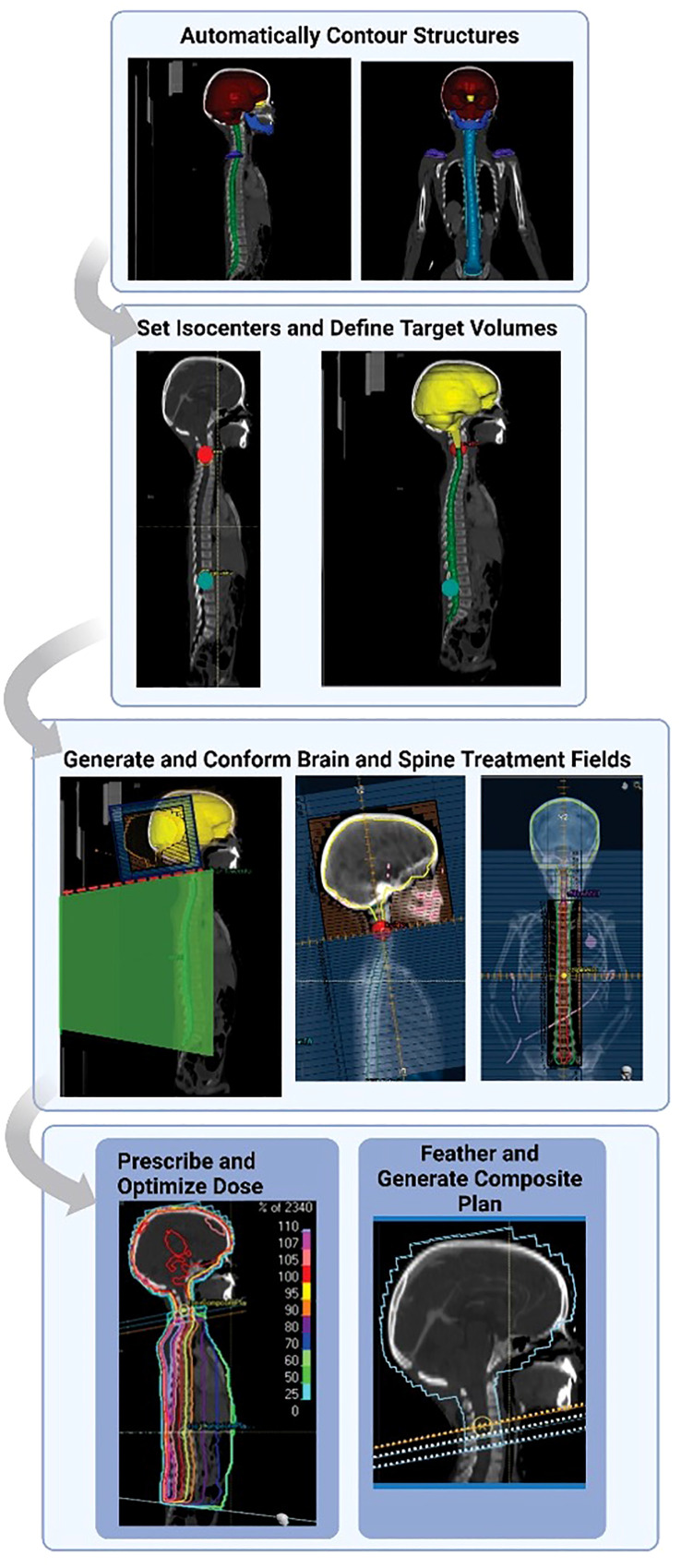
Outline of craniospinal irradiation auto-planning workflow. Normal structures and landmark structures are automatically contoured using deep learning methods. The autocontours then guide an autoplanning algorithm scripted in the treatment planning system. Auto-contours are used to automatically set isocenters and define target and prescription volumes. Fields are automatically generated and conformed to the specified targets. The dose is prescribed, and the dose to the spine field is optimized. The original plan is feathered with 2 junction shifts. Finally, a composite plan is generated. Figure reprinted from “Automating the treatment planning process for 3D-conformal pediatric craniospinal irradiation therapy,” by Hernandez et al., 2023, Pediatric Blood & Cancer, Volume 70(3), e30164. Copyright 2023 by John Wiley and Sons. Reprinted with permission.

Hernandez et al. previously automated the treatment planning process for 3D-conformal pediatric craniospinal radiation therapy (17). The algorithm was written in Raystation using the python-based API and did not use any auto-planning features native to the TPS. In summary (**Figure 1**), autocontours are first generated using previously-trained deep learning models and then they are imported into the treatment planning system. The autoplanning tool then generates 2 lateral brain fields (gantry at 90 and 270 degrees) matched to a single poster-anterior (PA) spine field (gantry at 180 degrees), an extended spine field (120 cm SSD to couch top), or 2 matched spine fields, depending on the patient’s spinal canal length. The MLCs for the brain and spine field(s) conform to a 1 cm uniform expansion of the brain autocontour and a 1 cm lateral expansion of the spinal canal autocontour, respectively. A half-beam block is implemented on the brain field to avoid the need for couch rotations. Spine subfields are then added and iteratively weighted to optimize the spine dose distribution. Finally, feathering is implemented at each match line to yield a composite treatment plan. All beam energies are set to 6 MV. The prescription is set to deliver 23.4 Gy in 13 fractions, normalized to give 95% of the prescribed dose to 100% of the brain volume and 95% of the spinal canal volume using a 5, 5, 3 fractionation scheme. For additional details on the contouring and planning algorithms, we refer the user to our previous work (17).

Prior to generating a treatment plan, the CSI autoplanning algorithm automatically performs a series of checks to ensure that the patient’s anatomy is compatible with the algorithm design. First, the algorithm automatically measures the patients’ spinal canal and determines whether to implement a single, extended, or multiple spine field configuration. In addition, the algorithm quantifies the amount of space available for junction shifts and decides to implement either 1- or 0.5-cm junction spacing. The algorithm will flag the user if there is <1 cm of space between the mandible and shoulders available for feathering. These patients were omitted from final testing. Finally, the algorithm automatically checks that the patient’s anatomy will be compatible with a half-beam block on the brain field by measuring the distance between the most superior slice of the brain contour and the most inferior slice of the mandible contour. A patient with a head tilt would have a higher mandible contour, which decreases the distance between the mandible and the top of the brain relative to that of a patient who is looking straight ahead. Patients with a measured brain-to-mandible distance larger than 20 cm were removed from the final testing set.

After removing the incompatible patients from the final testing set, we ran the autocontouring and autoplanning pipeline to generate CSI treatment plans. Plan quality was evaluated quantitatively with target coverage and dose to normal tissue metrics and qualitatively with physician review.

### Quantitative plan evaluation

To quantitatively evaluate the quality of the plans, dose metrics were analyzed across the final test set of patients. Target coverage was quantified using V95% of the prescription dose (23.4 Gy) evaluated for the brain, spinal canal, and cribriform plate. Normal tissue dose was also quantified using the maximum dose to the brain, spinal canal, brainstem, cochlea, eye, lens, and optic nerve autocontours. In addition, the mean dose was reported for the cochlea, heart, kidney, lacrimal gland, lung, pituitary gland, and thyroid autocontours.

### Qualitative plan evaluation

Physician review was used to evaluate the quality of the final autocontours and autoplans for each of the patients in the final testing cohort. Three pediatric radiation oncologists from 3 institutions (in the US and South Africa) reviewed the final test set. One patient was reviewed by all 3 physicians, resulting in a total of 53 plans for review. Each physician reviewed and scored each autocontour using a 5-point Likert scale detailed in **Table 1** (25). Using the same scale, the physicians reviewed the autoplan of each patient and assigned a clinical acceptability score to the brain and spine dose distributions individually. Autocontours and autoplans scored ≥3 was considered clinically acceptable. For plans that were scored as a 2, we also asked the physician if they would prefer to create their own plan from scratch or edit the plan we presented, as the original Likert scale did not have a metric for plans that required major edits but were still clinically useful.

**Table 1:** 5-Point Likert scale used to evaluate autocontour and autoplan quality (25).

Score	Acceptability	Description
5	Acceptable, use as-is	Clinically acceptable, could be used for treatment without any changes
4	Acceptable, minor and stylistic edits	Stylistic differences, but not clinically important
3	Acceptable, minor edits that are clinically necessary	Clinically important edits for which it is more efficient to edit the autocontours or autoplans than to start from scratch
2	Unacceptable, major edits	Edits that are required to ensure appropriate treatment and are significant enough that the user would prefer to start from scratch
1	Unacceptable, unusable	Autocontours or autoplans that are so bad that they are unusable (i.e. wrong body area or outside the confines of the body)

## Results

**Figure 2:**
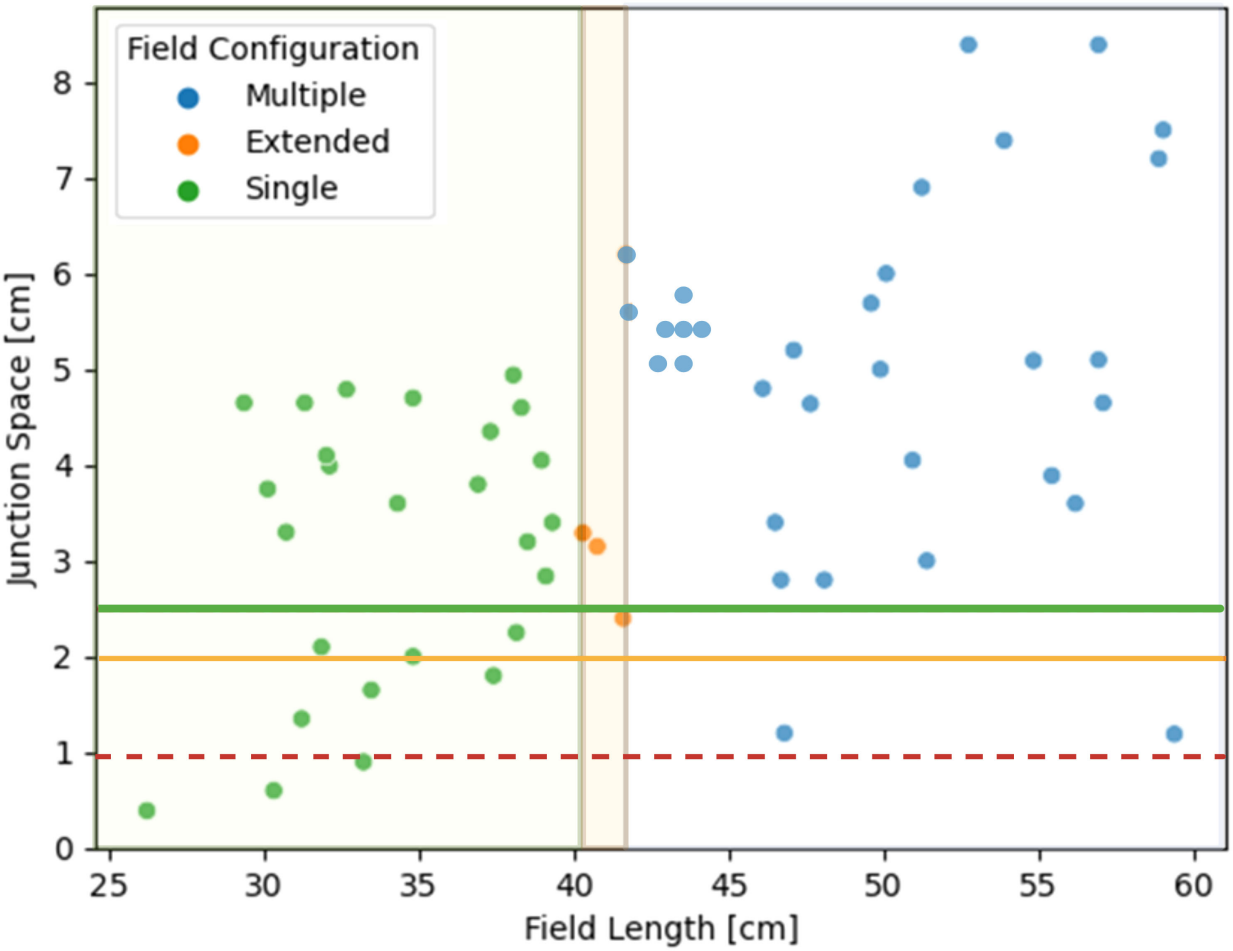
Distribution of available junction spacing and required spine field configurations for 63 patients. The green and yellow lines correspond to having enough feathering space for a 1-cm junction. The dotted red line represents the cut-off for a 0.5-cm junction.

81% (51/63) of patients met the autoplanning pre-processing requirements. Four patients were automatically removed for having less than 1 cm available to feather junctions and 8 patients were removed for not being compatible with a half-beam block on the brain field. Each flagged case was manually reviewed to verify that it was not compatible with the planning algorithm. **Figure 2** shows the variation in junction spacing and required spine field length measured across the dataset. A team of 3 pediatric radiation oncologists from different institutions reviewed and scored the resulting 51 autocontours and autoplans. One patient’s case was reviewed and scored by all 3 physicians (total of 53 plans scored). Physician 1 reviewed 16 plans, physician 2 reviewed 19, and physician 3 reviewed 18.

### Autocontouring

**Figure 3:**
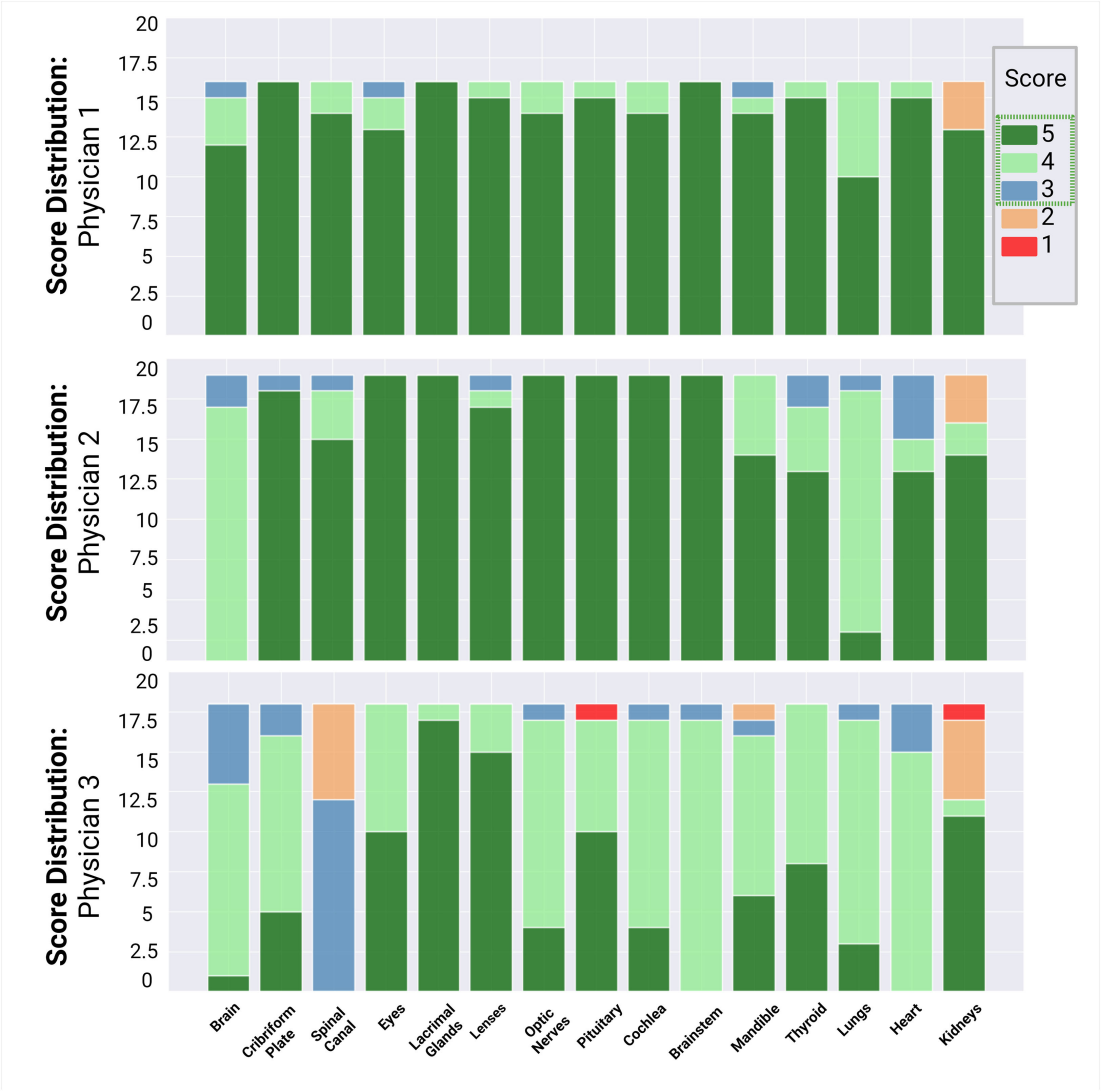
Summary of physician review of autocontours. Physicians 1, 2, and 3 reviewed and scored 240, 285, and 270 auto-contours, respectively. A score ≥3 (blue) was considered clinically acceptable.

Fifteen autocontours from 51 patients were reviewed by 3 pediatric radiation oncologists, including the eyes, lacrimal glands, lenses, cribriform plate, optic nerves, pituitary, cochlea, brainstem, mandible, brain, thyroid, lungs, heart, kidneys, and spinal canal. Physicians 1, 2, and 3 reviewed and scored 240, 285, and 270 autocontours, respectively. The scores assigned to each contour are summarized in **Figure 3**. Overall, the autocontouring model’s performance was robust across all spine field configurations. Across all physicians, 97% (775 of 795) of autocontours were scored as clinically acceptable, with 92% (733 of 795) of autocontours requiring no edits. Physicians 1, 2, and 3 scored 98%, 95%, and 85% of autocontours, respectively, as requiring no edits (score ≥4).

We evaluated the scores of the target autocontours (brain, cribriform plate, and spinal canal) and found that 85% (45 of 53) of the brain autocontours required no edits and the remaining 15% (8 of 53) required minor, clinically necessary edits because the temporal lobes and cribriform plate had been under contoured. All 51 cribriform plate contours were scored as clinically acceptable, and only 6% (3 of 53) required edits. Physicians 1 and 2 scored 100% of the reviewed spinal canal contours as clinically acceptable (score ≥3). Physician 3 scored 33% (6 of 18) of the spinal canal autocontours as clinically unacceptable (major edits required) because the canal contour was under contoured inferiorly and did not include the distal spinal nerve roots prior to exit from the ventral sacral foramina.

Normal tissue autosegmentation performed well for all structures but the kidneys due to variation in simulation planning technique. We found that 23% (12 of 53) of the kidney contours were scored as clinically unacceptable. The performance of the kidney autocontouring model was negatively affected by CT scans with contrast administered at the time of simulation. Because the autocontouring model was originally trained on non-contrast CT scans, the model was able to localize the kidneys but failed to accurately contour their shape, which resulted in major edits. The thyroid autocontouring model experienced a similar issue for one patient, when the model mistakenly assigned high-contrast vasculature near the thyroid as thyroid itself, which resulted in a minor, clinically necessary edit.

### Quantitative plan evaluation

**Figure 4:**
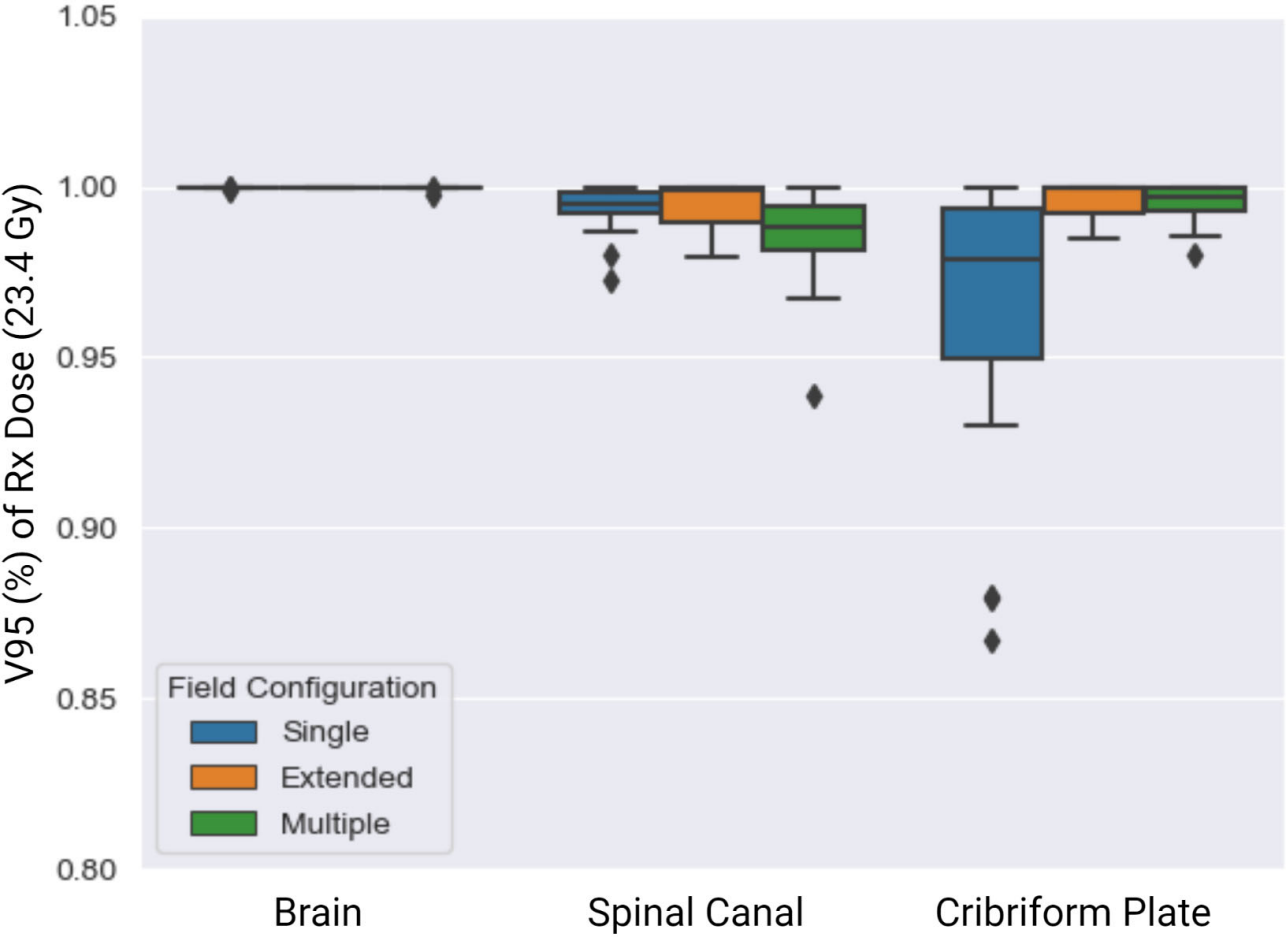
V95 (%) of Rx dose (23.4 Gy) across the single (blue), extended (orange), and multiple (green) spine field configurations. The brain field prescription was set to cover 100% of the brain with 95% of the prescription dose, which was achieved.

Of the 51 patients tested, 23, 3, and 25 required single-, extended-, and multiple-field configurations, respectively (**Figure 2**). The V95% achieved for the target structures across the single, extended, and multiple field configurations are summarized in **Figure 4**. The whole brain plan was normalized such that 100% of the brain autocontour received the prescribed dose, which was achieved across all three spine field configurations tested. The average V95 ± 1σ% for the spinal canal for single, extended, and multiple fields were 99.3 ± 0.04%, 99.3 ± 0.01%, and 98.6 ± 0.01%, respectively. Finally, the average V95% for the cribriform plate were 96.4 ± 0.01%, 99.5 ± 0.0002%, and 99.5 ± 0.01%, respectively.

The extended- and single-field configurations resulted in better target coverage to the spinal canal than did the multiple-field configuration. Finally, the extended- and multiple-field configurations achieved higher overall coverage to the cribriform plate than did the single-field configuration.

**Figure 5:**
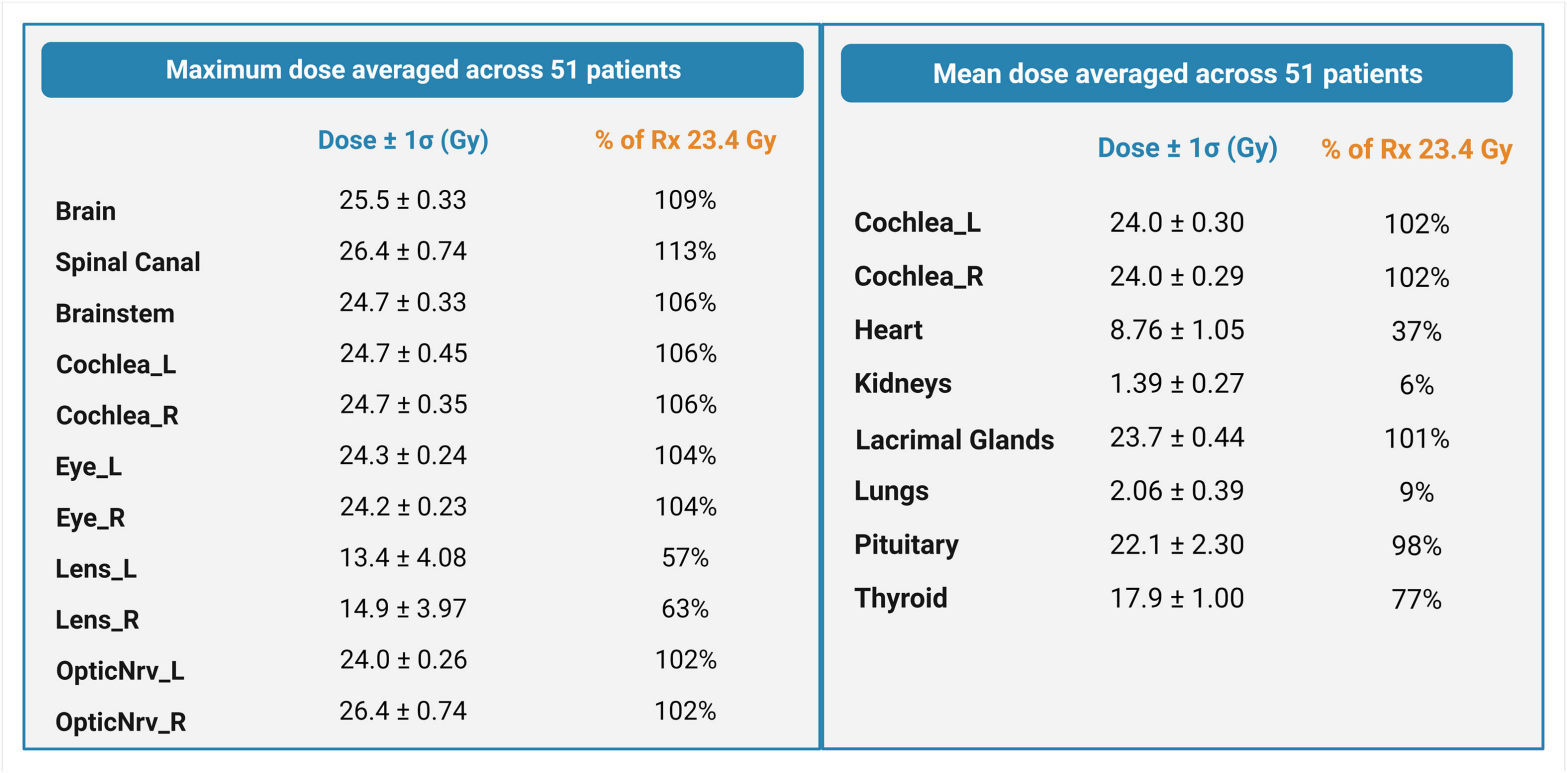
Maximum and mean doses averaged across 51 patients, expressed as a percentage of the prescription dose. Error estimates are standard deviations.

The average maximum doses (Gy) to the brain, spinal canal, and brainstem autocontours across all three spine field configurations were 25.5 ± 0.33 Gy (109% of Rx), 26.4 ± 0.74 Gy (113% of Rx), and 24.7 ± 0.33 Gy (106% of Rx), respectively. The dose to the spinal canal was higher for multiple-field plans (27.3 ± 0.37 Gy) than for single- and extended-field plans (25.6 ± 0.61 Gy and 25.5 ± 0.19 Gy, respectively). The average maximum doses (Gy) delivered to the cochlea, eye, lens, and nerve autocontours were 24.7 ± 0.40 Gy, 14.15 ± 4.03 Gy, and 25.2 ± 0.50 Gy, respectively (**Figure 5**). The mean doses [Gy] to the cochlea (L/R avg.), heart, kidney, lacrimal gland, lung, pituitary, and thyroid autocontours across all three spine field configurations were 24.0 ± 0.30 Gy, 8.76 ± 1.05 Gy, 1.39 ± 0.27 Gy, 23.7 ± 0.44 Gy, 2.06 ± 0.39 Gy, 22.1 ± 2.30 Gy, and 17.9 ± 1.00 Gy, respectively.

**Figure 6:**
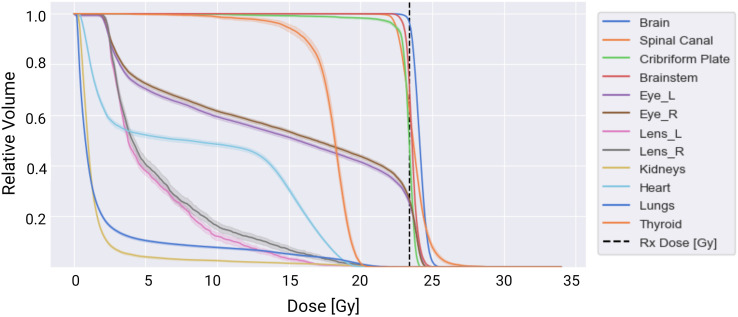
Dose-volume histogram summarizing dose delivered to the targets (brain, spinal canal, and cribriform plate) and normal tissues averaged across the 51 treatment plans tested. The solid lines represent the mean dose-volume histogram values, and the shaded portions represent one standard deviation in values across the three spine field configurations tested.

Overall, all spine field configurations resulted in consistent maximum and mean doses to the normal tissues. A dose-volume histogram for the target and normal tissue structures averaged across all spine configurations is summarized in **Figure 6**.

### Qualitative plan evaluation

**Figure 7:**
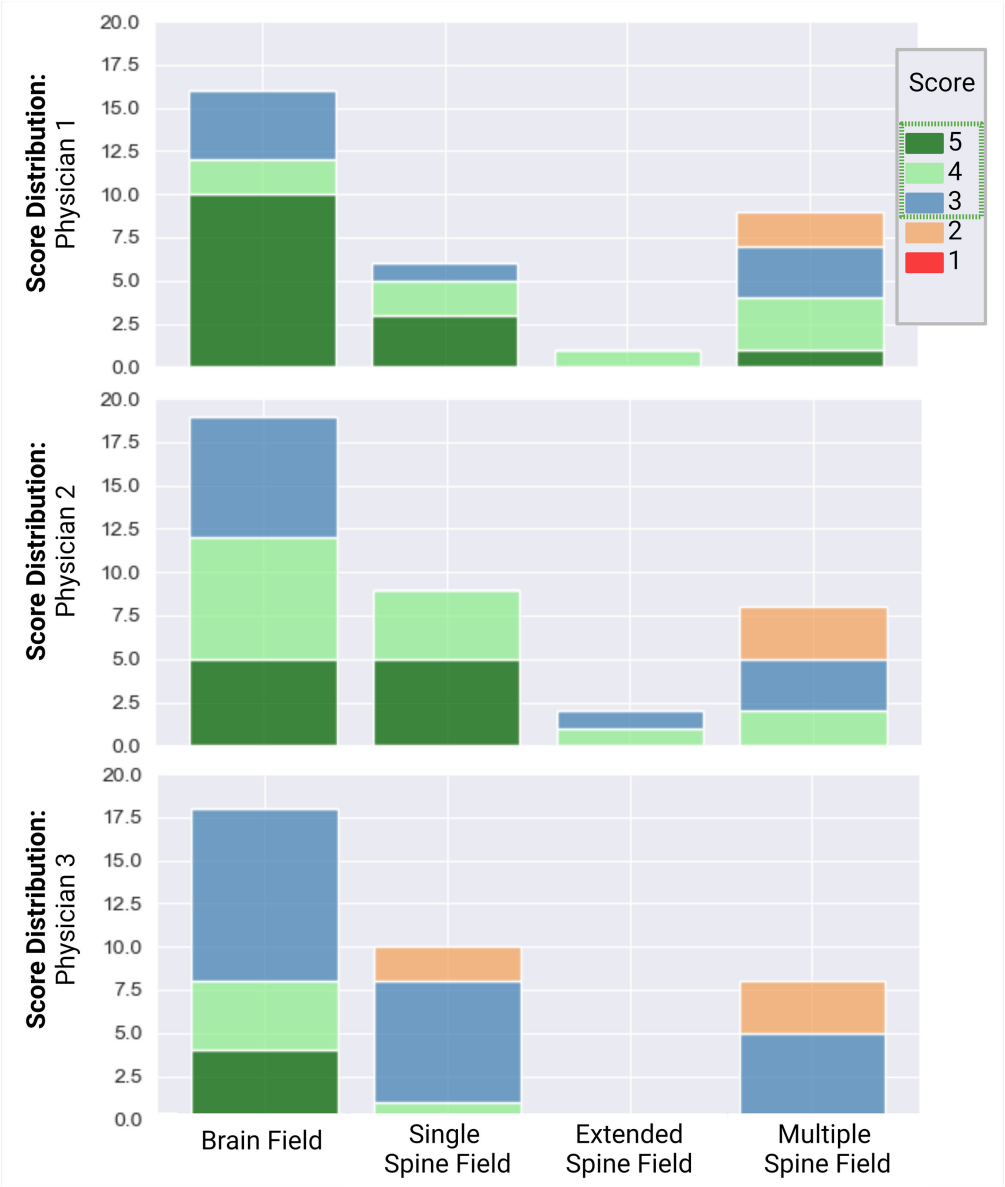
Scoring distribution for plans, reviewed by physicians 1, 2, and 3. Physician 1 reviewed 16 plans, physician 2 reviewed 19 plans, and physician 3 reviewed 18 plans. One single-field plan was reviewed by all 3 physicians. Individual scores were assigned to the brain and spine dose distributions. A score ≥3 (blue) was considered clinically acceptable.

A total of 51 patients were reviewed and scored for the quality of the composite CSI autoplan. One patient’s case was reviewed by all three physicians, resulting in a total of 53 plans. Physicians 1, 2, and 3 reviewed 16, 19, and 18 plans, respectively. For the single-field configuration, 6, 9, and 10 cases were reviewed by physicians 1, 2, and 3, respectively. For the extended-field configuration, 1 and 2 cases were reviewed by physicians 1 and 2, respectively. For the multiple-field configuration, 9, 8, and 8 cases were reviewed by physicians 1, 2, and 3, respectively. The scores of the autoplans from each physician are detailed in **Figure 7**. Factors contributing to the scores included the accuracy of the match lines; the dose distribution within the junctions; the target coverage to the brain, cribriform plate, and spinal canal; and the dose to normal tissues, such as the kidneys.

Overall, 100% of the brain dose distributions were scored as clinically acceptable (Likert score ≥3). Of these, 19, 13, and 21 were scored as 5, 4, and 3, respectively. For the spine dose distribution, 92% (23 of 25) of single-, 100% (3 of 3) of extended-, and 68% (17 of 25) of multiple-field cases were scored as clinically acceptable. Most plans required no edits or minor edits. Eight of the 25 multiple-field spine dose distributions were scored as clinically unacceptable, as they required major edits. However, all physicians reported that they would rather edit the autoplan rather than create a new one (**Figure 7**).

One plan was seen by all three physicians. Physicians 1, 2, and 3 assigned scores of 5, 4, and 3 to the brain dose distributions of the plan and 3, 4, and 4 to the spine dose distributions, demonstrating that while all physicians agreed that the plan was clinically acceptable, each had their own preference as to how they would edit the plan. Across all cases reviewed, all physicians agreed that the coverage to the cribriform plate could be improved on most of the plans, at the expense of an increased lens dose. The physicians had differing preferences on the tradeoff between spinal field coverage and hotspots.

Overall, the autoplanning algorithm worked well. The tool was able to generate composite treatment plans for 51 patients in three minutes per single-field case and eight minutes per multiple field cases. The additional time for multiple field cases was due to running optimization on the upper and lower spinal fields sequentially. It is important to note that the plan generation process does not require user intervention, yielding the potential for high clinical impact, particularly in resource-constrained centers.

## Discussion

We validated the performance of an autocontouring and autoplanning pipeline for craniospinal radiotherapy. The algorithms successfully generated 15 autocontours and a comprehensive CSI treatment plan for 51 patients across three spine field configurations. The performance of both tools was comprehensively analyzed using quantitative and qualitative metrics. The autocontouring model successfully generated clinically acceptable normal tissue contours and treatment plans, most of which required no or minor edits. While we observed inter physician variability on spine field scoring, all physicians commented that even if edits (major or minor) were required, they still preferred to edit our autoplans rather than create their own.

The autocontouring tool performed well for each of the 15 structures tested across 51 patients. Since the patients were anonymized prior to testing, we could not directly quantify how the models performed across different age groups. However, the spinal canal length for the 51 patients ranged from 25 cm to 60 cm; thus, we can infer that the model was robust to varying patient anatomy. The autocontouring model also worked well across varying image parameters. For example, the average slice thickness of the scans used to train the autocontouring models was 2.5 mm (1.25-2.5 mm range), and the average slice thickness of the scans from the external dataset was 1.5 mm.

All physicians scored all the brain autocontours as clinically acceptable. We found that the brain autocontouring model could be improved to increase temporal lobe coverage and accommodate patients with post-operative psudomeningoceles. Because the brain autocontour was generated by an adult autocontouring model, it had not been used on pediatric or postoperative cases before. While 2 physicians consistently scored the spinal canal autocontour as requiring no or only minor stylistic edits, one physician noted that the model consistently under contoured the nerve roots and scored the contours accordingly. This physician commented that 5-10 slices of the canal autocontour would require major edits but that it would still be more efficient to edit the autocontour than to create a new contour.

The physicians scored the majority of the normal tissue contours as requiring no or minor, stylistic edits, except for the lung and kidney autocontours. The lung autocontouring model consistently slightly under contoured the true lung volume, and the kidney model failed to accurately contour the kidneys on patients with contrast enhanced CT scans. Despite these errors, the physicians noted that the quality of the lung and kidney contours would not affect the final treatment plan.

Overall, the autoplanning tool performed well for the 51 patients tested across three spine field configurations. The scoring for the brain dose distribution was consistent across the three spine field configurations. The physicians noted that the brain dose distributions could be improved by increasing the cribriform plate coverage at the expense of increased lens dose, but this can be easily achieved by editing the position of the two or three MLCs that are shielding the lenses. Physicians noted that they would prefer to use additional brain sub-fields to reduce the size of the 107% hotspot. While our current CSI approach does not include sub-fields for the brain fields, they could easily be added using a technique that has been separately developed for whole brain radiation (15). Finally, one physician noted that the MLCs could be opened around the back of the skull to ensure that patients with pseudomeningoceles would be treated properly, with no negative effect on the patient.

For the spine field configurations, we found that the single- and extended-field configurations outperformed the multiple configuration plans. Ultimately, the validation of the algorithm proved that the multiple field configuration would need to be improved and further tested prior to clinical implementation. For many of the cases, the physicians were satisfied with the single-field spine dose distributions. They noted that they would adjust the weighting on the spine sub-fields to increase the spinal canal coverage at the expense of increasing the hotspot size. For the multiple-field cases, the match line between the upper and lower spine fields was designed to be placed just anterior to the spinal canal. This worked well for most patients; however, if a patient had an unusually angled spine, the first match point would be in the correct location and the match point for the latter 2 junctions would start to shift into the canal. The original algorithm was designed to add a single sub-field for single- and extended-field cases and 2 sub-fields for the upper and lower spine fields, respectively, for multiple-field cases. While this technique worked for most patients, physicians noted that they would add additional sub-fields to the multiple field plans to improve the plan quality. In addition, they could adjust the spacing of the spine sub-fields to optimize the dose distribution within the junction.

We identified limitations in our approach after testing it on patients from another institution. First, we encountered variations in clinical practice that the current algorithm was not designed to accommodate (i.e. the addition of sub-fields, patients with required brain fields >20 cm, or different prioritization of target coverage vs. hotspots). Another limitation was that it was not possible to validate our autocontours and autoplans with the clinical plans as we only received the anonymized CT scans and not the corresponding clinical contours and plans. In addition, the planning technique described in this work is currently limited to a single approach to CSI planning based on the recommendations from the SIOP PODC. We opted for 3D-conformal CSI planning as 84% of resource-constrained clinics report using this technique (6). Consequently, patients must have the appropriate setup to be treated with our technique (i.e. having the proper head tilt to achieve a half-beam brain block). Our pre-planning algorithm successfully identified 12 patients that were anatomically incompatible with the original planning design because of insufficient spacing between the mandible and shoulders for junction spacing, and/or insufficient head tilt to fit the brain into a half-beam block (20 cm). To expand the generalizability of our algorithm in the future, we plan to provide user training to ensure appropriate anatomical setup and accommodate couch kicks to treat larger brain fields.

Many institutions in HICs have moved to advanced techniques such as IMRT, VMAT or proton therapy for CSI. However, in LMICs, 3DCRT remains the prevalent technique, where this autoplanning tool would have the potential to produce high quality plans within a very short time. The autocontouring tool generates 15 normal tissue contours in 20 minutes and the autoplanning tool generates a comprehensive CSI plan in less than 3 minutes for the single field configurations and less than 8 minutes for the multiple field configuration. The process does not require any user intervention and both algorithms could be further optimized for time in the future. The efficiency of the tool has the potential to reduce contouring time and alleviate treatment delays which are known to be a major factor impacting survival (26). Additionally, the autocontouring tools are not specific to a single treatment technique or pediatric disease site; thus, they could affect all pediatric patients requiring radiation therapy. Such a tool could standardize contouring, helping to limit target deviations which impact treatment outcomes for both well-resourced and resource-constrained clinics (7, 27, 28).

The autocontouring and autoplanning tools described in this work will continue to go through rigorous testing before being implemented into the Radiation Planning Assistant. The RPA architecture has been proven to be robust to downtime, thus providing a reliable service to resource-constrained clinics (29). Finally, the RPA aims to provide autocontouring and autoplanning tools at minimal (most likely zero) cost to resource-constrained clinics in LMICs yielding potential for broad impact (16).

## Conclusions

In conclusion, we successfully validated an autoplanning pipeline developed at one institution using a large dataset provided by another institution. We automatically generated 15 normal tissue contours and a comprehensive CSI treatment plan for each patient without user intervention. The results indicate that our algorithm is robust in its adjustment to differing patient populations. Although the original algorithm was designed and tested exclusively in pediatric patients with medulloblastoma, we were able to successful generate treatment plans on a dataset that included a variety of disease sites requiring CSI, demonstrating that our algorithm is generalizable. Automating the contouring and planning workflow for pediatric CSI has the potential to increase treatment planning efficiency and global access to high-quality radiation therapy.

## Data availability statement

The raw data supporting the conclusions of this article will be made available by the authors, without undue reservation.

## Ethics statement

The studies involving humans were approved by MD Anderson Institutional Review Board. The studies were conducted in accordance with the local legislation and institutional requirements. Written informed consent for participation was not required from the participants or the participants’ legal guardians/next of kin in accordance with the national legislation and institutional requirements.

## Author contributions

All authors have made substantial contributions to the performance and analysis of the work, have helped draft or edit the manuscript, have provided approval of the submission, and have worked to ensure the accuracy of the results presented.
